# Nano-Molar Deltamethrin Sensor Based on Electrical Impedance of PAH/PAZO Layer-by-Layer Sensing Films

**DOI:** 10.3390/s130810167

**Published:** 2013-08-08

**Authors:** Luís Miguel Gomes Abegão, Jorge Humberto Fernandes Ribeiro, Paulo António Ribeiro, Maria Raposo

**Affiliations:** CEFITEC, Departamento de Física, Faculdade de Ciências e Tecnologia, FCT, Universidade Nova de Lisboa, Caparica 2829-516, Portugal

**Keywords:** deltamethrin, interdigital gold sensor, complex impedance spectroscopy, layer-by-layer, thin-film, PAH, PAZO

## Abstract

This work reports a novel deltamethrin (DM) sensor able to detect nano-molar concentrations in ethanol solutions. The sensing layer consists of a thin film, obtained via a layer-by-layer technique, from alternate adsorption of poly(allylamine chloride) (PAH) and poly[1-[4-(3-carboxy-4-hydroxyphenylazo)-benzenesulfonamide)-1,2-ethanediyl]sodium salt] (PAZO) onto a solid support with interdigitaded gold electrodes. The sensor response, obtained from impedance spectroscopy measurements, was revealed to be linear with respect to the real part of impedance, taken at 100 Hz, when plotted as a function of the logarithm of deltamethrin molar concentrations in the micro- to nano-molar range. Sensor sensitivity was of 41.1 ± 0.7 kΩ per decade of concentration for an immersion time above 2 min and the reproducibility is approximately 2% in a binary solution of ethanol and deltamethrin. The main insight of this work concerns to DM detection limits as the sensor revealed to be able to detect concentrations below 0.1 nM, a value which is significantly lower than any reported in the literature and close what is appropriate for *in situ* environmental contaminant detection.

## 1. Introduction

For the last decades there has been an exponential growth in the application of pesticides, insecticides, herbicides and bactericides in agricultural activities, due to demand of food production as a result of the World demography increase. These products, known as plant protection products (PPP), contain active toxic substances (AS) that may contaminate agricultural products, at harvest or storage after treatment, in processed products and in the surrounding crops areas as well [1–3]. In fact, the widespread use of crop protection chemicals in agricultural production generates the contamination of adjacent soil and aquifers in such a way that even the so called “organic food” is hardly free of AS residues. Thus, the measuring and monitoring in situ the concentration of AS residues is a relevant issue to be addressed for the sake of human health [4].

From the point of view of chemical sensing devices referred in the literature and used for this purpose, they normally use transduction techniques such as potentiometry, amperometry or cyclic voltametry. Potentiometry operates in terms of the system net charge, being disadvantageous when applied to non-electrolyte media, whereas cyclic voltametry operates in solution requiring compounds that can be oxidized or reduced actively onto a work electrode [5,6]. Generally, these techniques allow the detection of individual compounds or subgroups of related pollutants, in binary aqueous solutions samples. However, water “samples” taken from soil or aquifers usually consist of a complex matrix of different compounds [7,8]. In this way a sensor device with both classification and quantifying capabilities will be the most adequate for this purpose. These features can be found in the so-called electronic tongues which are particularly adequate for liquids analysis and classification. The analogy with the biological system [9] suggests that the “electronic tongue” is based in the taste recognition and not in the discrimination of individual chemical substances. The main advantage of these devices is the capability of continuous monitoring and the detection of analytes with concentrations below of the perception biological limits [10]. These devices normally use a set of sensorial units not specific for determined chemical species, but give a response pattern which is a fingerprint of the characteristics or qualities of the analyzed sample. Recently, a new type of “electronic tongue” was developed using thin film layers of conducting polymers and lipids as sensorial units and the electric impedance was used to translate the sensors response [11]. This system revealed to be able of recognizing the taste below the biological limits and was used in the evaluation of the quality of several types of beverages in classes in accordance with taste, allowing intensity quantification as well as detection of non-electrolytic substances [12–14]. In this case the capacitive part of electrical impedance at, the frequency of 1 kHz [13], was used to describe the sensor response with quantification and classification carried out through the component principal analysis (PCA), neural networks or complex networks theories [15,16]. The production of thin layers of active molecules to be used as sensing sensor part can be achieved by the layer-by-layer (LBL) technique [17–19], which consists of the alternate adsorption from solution of positive and negative polyelectrolyte molecules. This technique presents several advantages, as it allows precise control of film thicknesses and architecture, and is consistent with dedicated patterning techniques which includes microcontacting printing, conventional photolithography, photochemistry and chemical selectivity [20,21], thus allowing that molecules to be adsorbed onto a determined spatial region or pattern. These concepts can be used for the development of lab-on-a-chip sensors, which is a relevant issue for development of *in situ* sensors. Furthermore, the LBL technique has been used to assemble a wide variety of molecules such as polyelectrolytes, ceramics, nanotubes and biological molecules as DNA, proteins, enzymes and lipids, thus offering a large diversity of possibilities for the buildup of AS sensing layers.

In this article, a sensing layer, based on LbL films prepared with the common polyelectrolyte poly(allylamine hydrochloride) (PAH) and azo-polyectrolyte poly[1-[4-(3-carboxy-4-hydroxyphenyl-azo) benzenesulfonamido]-1,2-ethanediyl, sodium salt] (PAZO), is proposed for the detection of delthametrin (DM). Delthametrin is a pyrethroid pesticide, belonging to the PPP insecticides group and being the most commonly AS used in the history of agriculture and extremely toxic for fish [22] and zooplankton communities [23]. This pesticide has been proven to alter the development of the larvae of oysters [24] and the filtration and pumping activity of freshwater mussel [25]. The modifying effect of DM on sodium and potassium channels has also been demonstrated in molluscan neurons [26]. The adverse effects of DM on the environment together with its intensive use requires that efforts be undertaken towards the development of low concentration DM sensors for *in situ* environmental monitoring. To address this issue, a sensing layer based on a PAZO thin film was implemented and impedance measurements used for signal transduction. The reason for using PAZO polyelectrolyte is that its azo group-based chemical structure is akin to that of DM, in the sense that they can adsorb through physical interactions. In addition, PAZO is known to be easily assembled in an LBL film form, namely with PAH polyelectrolyte, with all of adsorption kinetics, layers growth and morphology well characterized [27–30]. Thus, in order to investigate the feasibility of this approach, PAH/PAZO LbL films were prepared onto solid glass supports having interdigitated electrodes and both resistance and capacitance of the equivalent circuit which is created measured when the electrodes were immersed in solutions with DM analyte.

## 2. Experimental Section

**Figure 1.**
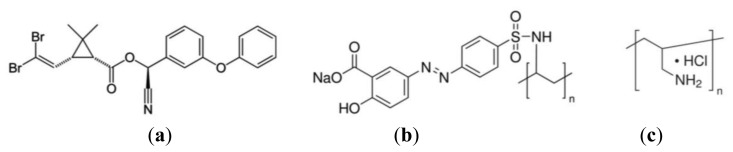
Chemical Structures of (**a**) Deltamethrin; (**b**) PAZO and (**c**) PAH.

DM [C_22_H_19_Br_2_NO_3_, Figure 1a], was purchased in analytical solution from Sigma-Aldrich (St. Louis, MO, USA). The sensing layer consisted of LBL films prepared with the polyelectrolytes poly[1-[4-(3-carboxy-4-hydroxyphenylazo)benzenesulfonamide]-1,2-ethanediyl, sodium salt] (PAZO**)** and poly(allylamine hydrochloride) (PAH), obtained from Aldrich and with the chemical structures shown in Figure 1b and c. The reason for choosing those polyelectrolytes is that their assembly characteristics are well known, namely the growth characteristics and adsorption kinetics [27–30] and PAZO presents two benzene rings—Figure 1a), which are likely to interact with benzene rings of the DM molecule. Relatively to the films' structure, it should be added that as the PAH/PAZO LBL films are obtained by alternated adsorption of layers, the structure of these films can be defined by a sequence of more or less planar bilayers. Since the adsorbed amount per unit of area and per bilayer is constant, the thickness of each bilayer will be also constant. However, the LBL films structure is also influenced by the surface morphology of each layer. For the first five bilayers, it was seen that the surface roughness increases slightly with the number of bilayers [30], which indicates that all bilayers are essentially similar without increase of larger grains and consequently the bilayers are more or less planar with similar values of thickness [31] and roughness [30]. Since the film structure, namely, the thickness and roughness, is also relevant for the sensor response, and being the film composed by a set of bilayers, the resistance and capacitance values of the sensing film can be controlled by the number of bilayers. For the present case, five bilayer films were implemented because they present an acceptable value of resistance, not too small or too large, allowing detection of the analyte over a range of different concentrations.

For the LBL film preparation, polyelectrolyte aqueous solutions were prepared using pure water (resistivity of 18 MΩcm) supplied by a Milli-Q system from Millipore (Billerica, MA, USA). The PAZO polyelectrolyte was dissolved in to a monomeric concentration of 10^−2^ M with pH of *ca.* 9 and PAH aqueous solutions were 10^−2^ M concentration and a pH that was set by adding either HCl or NaOH. The PAH/PAZO LBL films with five bilayers, (PAH/PAZO)_5_, were adsorbed on gold interdigitated electrodes deposited onto glass supports. These electrodes consist of 12 digits, each 6 mm long, 0.25 mm width, 0.1 μm high and 0.35 mm apart from each other. The electrode was fabricated by evaporating gold onto a BK7 glass substrate with a primer bonding layer of chromium and using a photolithographic method to create the fingers pattern [11].

Deposition comprised sequential immersion of the solid support in the PAH and PAZO polyelectrolytes solutions during 45 seconds with a 3 seconds rising step in ultrapure water in between. This procedure was repeated five times to create a live bilayers (PAH/PAZO)_5_ thin film, with the last layer being of PAZO. The adsorption time of polyelectrolytes were chosen in accordance with Ferreira *et al.* [27]. As DM has a poor solubility in water (<0.2 μg·L^−1^), ethanol solutions of DM with concentrations of 10^−6^ M to 10^−10^ M were prepared.

The complex electrical impedance *Z* = *R* ± *iC*, where R is the resistance and C is the capacitance, of the sensorial layer when immersed in each DM solutions were measured using a model HM8118 impedance analyzer (Hameg, Mainhausen, Germany) in the frequency range of 20 Hz to 200 kHz with a driving voltage of 1 V. The impedance values were measured after different periods of time of sensor immersion in the DM solution. These periods of time ranged from 2 s to 4 min in accordance with the performed experiment and are indicated when necessary in the results section.

The PAH/PAZO LBL films were also characterized by ultraviolet-visible (UV-Vis) region using a model Evolution 300 spectrophotometer (Thermo Scientific, Waltham, MA, USA) and the adsorbed amount of DM adsorbed onto (PAH/PAZO)_5_ films were measured using a model QCM200 Quartz Crystal Microbalance (QCM, Stanford Research Systems, Sunnyvale, CA, USA).

## 3. Results and Discussion

**Figure 2.**
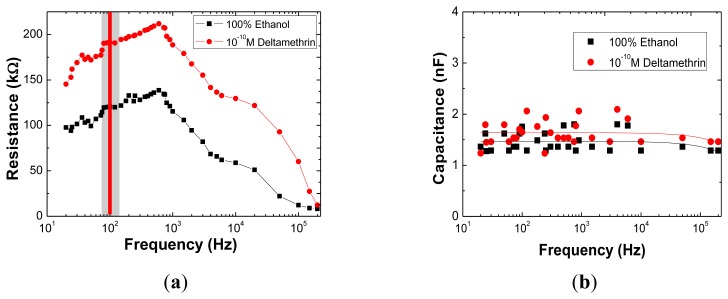
Electrical resistance (**a**) and capacitance; (**b**) of impedance complex spectra obtained when the sensor is immersed in ethanol and a DM concentration of 10^−10^ M. Solid lines are just guide lines to the eye.

Results for the complex electrical impedance measurements in the range of 20 Hz to 200 kHz are shown in Figure 2a and b, where electrical resistance and capacitance are plotted as a function of frequency for the sensor immersed in ethanol pure solution and in 10^−10^ M DM solution. These spectra correspond to the resistance and capacitance of the equivalent electrical circuit [13,32,33] which includes all interfaces, namely, the insulating or blocking layer associated to the (PAH/PAZO)_5_ LBL film, the double layer created and the bulk electrolyte. The decomposition of the spectra into its electrical components is only justified if spectra peaks are changing with the analyte concentration. In the present case, the spectra show that the R values for both trends, obtained under these conditions in the frequency range of 80 to 120 Hz (Figure 2a) show a plateau, which is therefore the best frequency range for evaluating a single point difference between the curves. This difference is of approximately 70 kΩ. In the same frequency range, and actually at all scanned frequencies, no difference between blank and 0.1 nM DM solution appears instead for the C signal, see Figure 2b. Thus the sensor system is able to detect DM in very low concentrations as the R is distinguishing this range of DM concentrations.

In order to check if Z values are influenced by the immersion time of the sensor in the DM solution, the frequency was fixed at 100 Hz and Z was recorded after different immersion times. This procedure was repeated for different DM concentrations in a range of 10^−10^ to 10^−6^ M and for 0 M concentration. These obtained R and C data are shown in Figure 2a and b, respectively. These curves are related with the adsorption of the DM molecules onto the sensor surface which contributes to changes of the electrical resistance associated with the insulating or blocking layer of the sensor-sensing layer or sensing layer+DM adsorbed molecules. As the DM adsorption phenomena is a time function, both electrical components are time dependent.

**Figure 3.**
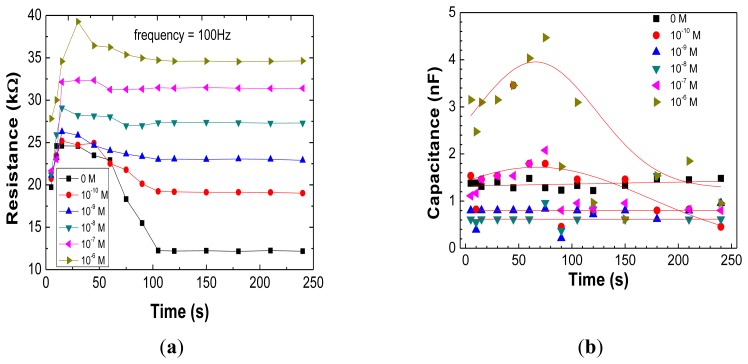
Immersion time analysis of Z at a fixed frequency, 100 Hz: (**a**) electrical resistance; (**b**) capacitance, for different DM concentrations. Solid lines are just guide lines to the eye.

By analyzing Figure 3a one can infer that the sensor resistance is stable for immersion times greater than 2 min, indicating that the impedance spectra should be measured at immersion times higher than this value, for the sake of data reproducibility. Whereas in Figure 2b it appears that there was no difference in C between blank and 0.1 M DM solution for the 2 min soaking time sample, from Figure 3b the C signal is clearly able to discriminate between DM concentrations, when the sample soaking time is kept at shorter values of less than 75 s. On the contrary the R is less sensitive, see values in Figure 3a at e.g., 1 min, vertical intercept line.

**Figure 4.**
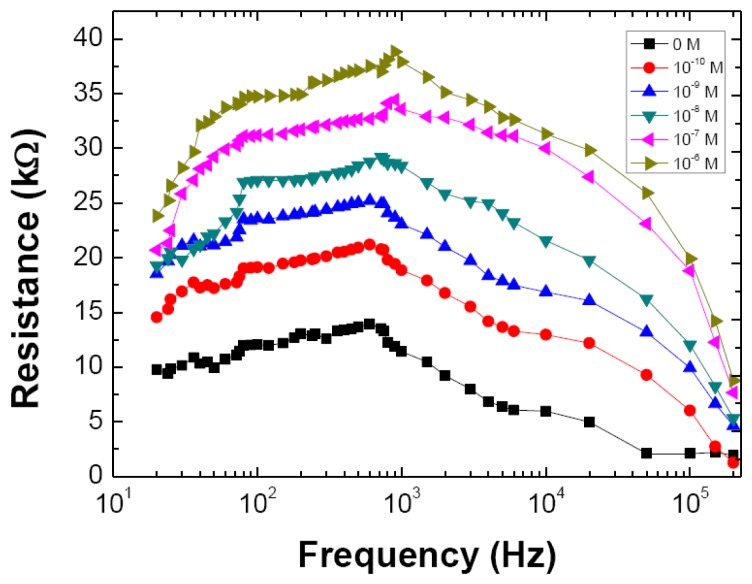
Electrical resistance of Z spectra obtained when the sensor is immersed on different DM concentrations and left at rest for 2.5 min in each solution before spectrum measurement. Solid lines are just guide lines to the eye.

Thus, either R or C, could be used to realize a DM sensor. Nevertheless it was decided to remain with R as it will be easier to implement the sensor in a purely resistive measurement system, which will make a possible commercial device less expensive. Accordingly, the R was measured in the 20 Hz to 200 kHz frequency range as a function of DM concentration, after the sensor system being left at rest in each solution for exactly 2.5 min, which is a time well within the time window where the response has already reached the constant, saturated flat level, which happens after approximately 2 min. The results are shown in Figure 4, where once more the resistive part of impedance curves reveal similar behavior to those displayed in Figure 2, with the resistance plateau, particularly in the 80 Hz to 120 Hz region, stable at all concentration levels.

**Figure 5.**
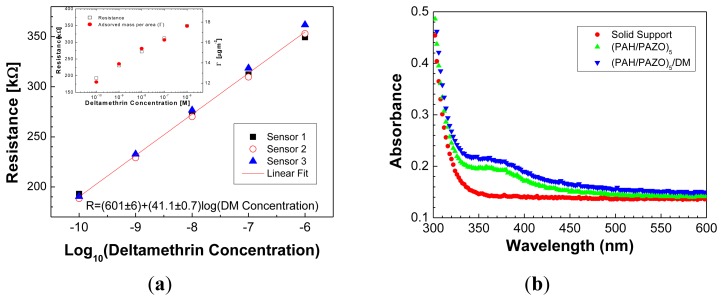
(**a**) Electrical resistance at 100 Hz as a function of logarithm of DM concentration obtained for different sensor systems. The solid line is the fit of experimental data to a straight line. The inset shows both the electrical resistance at 100 Hz and adsorbed amount per unit of area as a function of DM concentration; (**b**) UV-visible spectra of (PAH/PAZO)_5_ LbL film before and after DM adsorption.

If one plots the resistance values measured at 100 Hz, Figure 5a, as a function of DM solution concentration, a nonlinear increase is observed. The 0 M data was not included in Figure 5a due to the logarithmic scale used. When plotting the resistance as a function of logarithm of concentration, a straight line is clearly drawn, allowing one to determine a sensitivity of 41.1 ± 0.7 kΩ per decade of molar concentration in the considered range.

This value is an average obtained from three different sensing layers prepared under similar conditions. The standard deviation of the resistance measured for the data points of Figure 5 is approximately 2%, which demonstrates a high reproducibility of our sensor. Concerning the sensor resolution, we are interested in achieving the highest possible value of sensitivity for the sensor to be sensitive to small changes in analyte concentration. As in the present case the sensitivity is the resistance change per unit of log of concentration, one can scale each concentration decade in parts of 0.02, value which can be obtained dividing a concentration decade by 41.1 kΩ/0.7 kΩ ≈ 59, 1/59 ≈ 0.02. This leads to a sensor resolution of 5 pM for the smallest concentration used, 0.1 nM. Finally, it should be mentioned that the detection limit is lower than 0.1 nM which is much lower that obtained, for example, by Ge *et al.* [34].

In order to relate the results of electrical resistance with DM uptake by the LBL sensing films, the amount of DM adsorbed per unit of area onto (PAH/PAZO)_5_ films was measured with a QCM, for different DM concentrations and adsorption times of 150 s. For this experiment, (PAH/PAZO)_5_ films were prepared on a QCM crystal quartz and the frequency shifts associated to the DM adsorbed amount per unit of area were measured after immersion of the QCM crystal in DM solutions with different concentrations. It should be indicated that one expects that the number of adsorbed DM molecules to be also dependent on the number of bilayers of the LBL film. In the present case, the sensor was based in a fixed number of bilayers and in the DM adsorption experiments a film with the same number of bilayers was prepared on the QCM support, before DM adsorption characterization in order to have similar conditions. A linear increase of DM adsorbed amount per unit of area is observed when plotted as a function of log of DM molar concentration, see inset of Figure 5a. As R and adsorbed amount per unit of area with the log of DM concentration follow the same trend, one can conclude that resistance response is related with DM adsorption which made the interface more thick and insulating. The presence of adsorbed DM on the sensing layer was also inferred by comparing the UV-Vis spectra of a (PAH/PAZO)_5_ film before and after DM adsorption, see Figure 5b. The spectra show that although the baseline to be shifted due to the adsorption of the colorless DM molecules, the absorbance intensity of the azo chromophore absorbance peak at 360 nm [31] remains constant.

## 4. Conclusions

LBL films of PAH/PAZO were revealed to be suitable as sensorial layers in a DM sensor device for DM detection in ethanol solutions in the concentration range of 10^−10^ M to 10^−6^ M. Electrical resistance measurement at 100 Hz, were revealed to be an adequate variable for DM concentration transduction, as it presents a linear behavior which gives rise to a sensitivity of 41.1 ± 0.7 kΩ per decade of concentration and good reproducibility.
